# Association between endometrial indoleamine 2,3-dioxygenase expression level and pregnancy outcomes in women undergoing first in vitro fertilization treatment

**DOI:** 10.1186/s12884-020-03511-9

**Published:** 2021-01-07

**Authors:** Su Liu, Ling Hong, Yuye Li, Ruochun Lian, Xiaohui Wang, Yong Zeng

**Affiliations:** Shenzhen Key Laboratory of Reproductive Immunology for Peri-implantation, Shenzhen Zhongshan Institute for Reproduction and Genetics, Fertility Center, Shenzhen Zhongshan Urology Hospital, No.1001 Fuqiang Road Futian District, 518045 Shenzhen, China

**Keywords:** Indoleamine 2,3-dioxygenase, Endometrium, In vitro fertilization, Live birth

## Abstract

**Background:**

Indoleamine 2,3-dioxygenase (IDO) has been reported to play a key role in placental development during normal pregnancy. However, the question of whether endometrial IDO expression affects in vitro fertilization (IVF) pregnancy outcomes remains unclear. The current study was undertaken to investigate whether there was any association between endometrial IDO immunohistochemical staining and IVF treatment outcome.

**Methods:**

This retrospective study was designed to compare pregnancy outcomes among women with different endometrial IDO expression levels under their first IVF treatment. A total of 140 women undergoing their IVF treatment were selected from January 2017 to December 2017. Endometrial samples were collected during mid-luteal phase before IVF cycle. The endometrial IDO expression levels were analyzed by immunohistochemistry, and compared between women who were pregnant or not. A logistic regression analysis was performed to determine the impact of endometrial IDO staining on live birth.

**Results:**

No significant differences in the endometrial IDO immunohistochemical staining were found between women who had clinical pregnancy and those who failed (*P*>0.05). However, the endometrial IDO staining was significantly higher among women who had live birth compared with those who had no live birth (*P*=0.031). Additionally, after adjusting for differences in maternal age, BMI and duration of gonadotropin stimulation, women with higher IDO expression level had an increased live birth rate (adjusted odds ratio [aOR] 2.863, 95% confidence interval [CI] 1.180-6.947).

**Conclusions:**

Higher endometrial IDO expression level during mid-luteal phase is associated with an increased live birth rate in women undergoing their first IVF treatment.

## Background

Infertility, which was defined as failure to establish a successful pregnancy after 12 months of unprotected intercourse or therapeutic donor insemination, affects approximately 1 to 2 hundred million couples of reproductive age worldwide [1, 2]. In vitro fertilization and embryo transfer (IVF-ET) has developed as the primary choice to treat infertility associated with male factors, endometriosis, ovarian dysfunction and other unexplained reasons.

Numerous studies have provided better comprehension of mechanisms that influence fertilization and implantation, however, it still remains a major challenge to find proper biomarkers that may predict pregnancy issues after IVF, which can be assessed before the initiation of treatment. The process of implantation and the maintenance of normal pregnancy may include mechanisms preventing rejection of the allogeneic fetus. A proportion of studies postulated that the abnormal pregnancy outcomes might be due to immunological dysfunction, but the precise mechanisms for the disturbance of immune tolerance at the fetal-maternal interface are still poorly understood. To maintain normal pregnancy, the immune system is tightly regulated and in part by metabolic pathways [3].

As part of a network of “metabolic immune regulation”, indoleamine 2,3-dioxygenase (IDO) has been reported to play a key role in placental development during normal pregnancy [4, 5]. IDO has diverse biological roles. Firstly, IDO catalyzes the initial and rate-limiting step of tryptophan degradation along the kynurenine pathway, produces immune modulatory tryptophan metabolites, and therefore exhibits immunosuppressive effects [6, 7]. The IDO pathway contributes to Treg differentiation and activation of functional suppressor activity in mature Tregs [8, 9]. Initial evidence for IDO-mediated immunosuppression was demonstrated at the maternal-fetal interface [10]. Inhibition of IDO with the competitive inhibitor results in the rejection of allogeneic fetuses, supporting the role of IDO as a negative regulator of immunity in vivo [10]. Secondly, IDO-induced tryptophan degradation could eliminate invading pathogens by nutriment competition [11]. Thirdly, IDO is also involved in the efficient invasion of the endometrial tissue by trophoblastic cells, which is a prerequisite for the occurrence of a normal pregnancy [12, 13]. These observations suggest that although IDO shares its role with other molecular mediators, it is a key element controlling normal pregnancy.

Sex hormones are important component of microenvironment of endometrium and maternal-fetal interface. IDO mRNA expression level and its metabolite kynurenine could be increased in lipopolysaccharide (LPS)- and IFN-γ-stimulated bone marrow-derived DC by hCG treatment [14]. In addition, estrogen treatment could upregulate IDO expression in stromal cells and macrophages in endometrium [15]. Unlike hCG and estrogen, IDO expression is suppressed in progesterone-conditioned endometrial stromal cells [16, 17]. Thus, IDO participates as part of a network of endocrine and immune system and helps create conditions that favor the maintenance of normal pregnancy.

Accumulating evidences indicate that reduced activity or expression level of IDO may lead to pathological pregnancies. Our previous study showed that the endometrial IDO expression level in women with recurrent miscarriage (RM) was significantly lower than that of normal fertile controls [18]. In term placenta, decreased IDO expression was found to be associated with the severity and onset time of pre-eclampsia [19]. However, the question of whether endometrial IDO expression affects IVF pregnancy outcomes remains unclear. Thus, we investigated IDO protein expression in the mid-luteal phase when endometrium is prepared for implantation and retrospectively analyzed the possible correlation of endometrial IDO expression with characteristics of infertile patients and pregnancy outcomes. The contribution of endometrial IDO expression to live birth was further determined using logistic regression analysis.

## Methods

### Participants

**Fig. 1:**
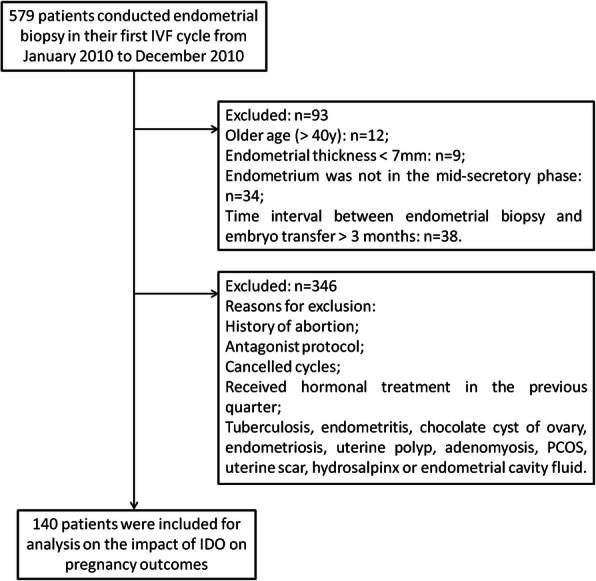
Flowchart of the present study

This was a retrospective cohort study of 140 infertile women who underwent their first IVF cycle in the Fertility Center, Shenzhen Zhongshan Urology Hospital (SZUH) from January 2017 to December 2017. As demonstrated in Fig. 1, patients were included if they met the following criteria: (1) age ≤ 40 years old; (2) agreed to conduct endometrial biopsy before controlled ovarian hyperstimulation (COH); (3) the endometrial thickness ≥ 7 mm on the day of biopsy; (4) the endometrium was in the mid-secretory phase confirmed by hematoxylin and eosin (H&E) staining; (5) the time interval between endometrial biopsy and embryo transfer was less than 3 months. Patients were excluded if they had: (1) history of medical or surgical abortion; (2) history of spontaneous abortion; (3) history of IVF-ET treatment; (4) the following adverse factors in the uterine cavity: tuberculosis, endometritis, chocolate cyst of ovary, endometriosis, uterine polyp, adenomyosis, polycystic ovary syndrome (PCOS), uterine scar, hydrosalpinx or endometrial cavity fluid; (5) treated with antagonist protocols. Only patients treated with GnRH agonist long protocol were included in this study. No subjects had received hormonal treatment in the previous quarter. This retrospective study was approved by the reproductive research ethics committees of SZUH (Approval number: SZZSECHU-F-20,019,037). Informed consent was provided by all subjects at recruitment.

### Immunohistochemical and imaging analysis

For patients in the natural cycle group, endometrial tissues were collected using an endometrial curette after 7–9 days of LH surge. For patients in the hormone replacement cycle group, endometrial tissues were collected after 7–9 days of administration of progesterone. After washing with phosphatebuffered saline (PBS), the endometrial samples were fixed with 4% paraformaldehyde 6 to 12 h at room temperature. Then, all samples were processed into paraffin within 48 h. Paraffin-embedded endometrial tissues were cut into 4-µm sections followed by deparaffinization and dehydration for immunohistochemistry. The histologic phase was confirmed by H&E staining. IHC staining procedure was performed using BOND-III Fully Automated IHC and ISH Stainer according to the manufacturer’s instructions (Leica Biosystems, Wetzlar, Germany). The primary antibody targeted to IDO was purchased from Cell Signaling Technology, Danvers, Massachusetts (catalog number: 86,630; dilution: 1:300). Other reagents for IHC were provided by Bond™ Polymer Refine Detection (Catalog No: DS9800) kit. For IDO, the staining was graded by 2 observers independently based on the staining intensity at a magnification of 200 × field (0.95 mm^2^). Grade 1, no cells were stained or stained weakly and scarcely positive; grade 2, the cells were stained moderately and focally positive; grade 3, the cells were stained strongly and diffusely positive [18, 19]. The staining grade was used to a mix of all stained cells.

### IVF and embryo transfer protocols

All included patients received a routine luteal phase down-regulation protocol with GnRH agonist protocol. The gonadotropin starting dose and the GnRH analogue were selected based on the physician’s discretion. Final oocyte maturation was induced by injection of human chorionic gonadotropin (hCG) when at least 2 follicles had reached a mean diameter of 18 mm as observed on ultrasound scan. The serum E_2_ concentration and endometrial thickness were measured on the day of hCG administration. Oocyte retrieval was carried out 36 h after ovulation trigger by transvaginal ultrasonographically guided needle aspiration. The oocytes were fertilized by traditional IVF or intracytoplasmic sperm injection (ICSI), according to the oocyte and semen status. Embryo quality was evaluated by 2 experienced embryologists based on the number of blastomeres as well as the percentage of fragmentation. Cleavage-stage embryos or blastocysts with good morphological quality were chosen for embryo transfer on day 3 or day 5 after fertilization.

### Outcome measures

The pregnancy outcomes in this study included the implantation, clinical pregnancy, miscarriage, and live birth rates. The implantation rate was calculated as number of intrauterine gestational sacs per total number of transferred embryos. Clinical pregnancy was defined as the observation of a gestational sac under ultrasonography 4 to 5 weeks after embryo transfer. Miscarriage was defined as fetus loss within the first 12 gestational weeks. Live birth rate was calculated as cycles with delivered live babies per embryo transfer cycle.

### Statistical analysis

Descriptive statistical analysis was performed on main maternal and cycle characteristics. Continuous data with normal distribution were presented as the mean ± standard deviation (SD) and analyzed by independent *t*-test. The continuous variables that did not show a normal distribution were presented as median and interquartile range, and were analyzed by Mann-Whitney *U*-test. Categorical data were presented by the number of cases and corresponding percentage, and were analyzed by Pearson’s χ^2^ test or Fisher’s exact test. To assess the relationships between endometrial IDO expression levels and different pregnancy outcomes, logistic regression analysis, adjusted for maternal age and BMI, were performed. All *P* values are 2 sided and statistical significance was established as *P* < 0.05. All analyses were conducted using SPSS (version 23.0; SPSS Inc.).

## Results

### Immunohistochemical grading of IDO expression in the endometrium

**Fig. 2:**
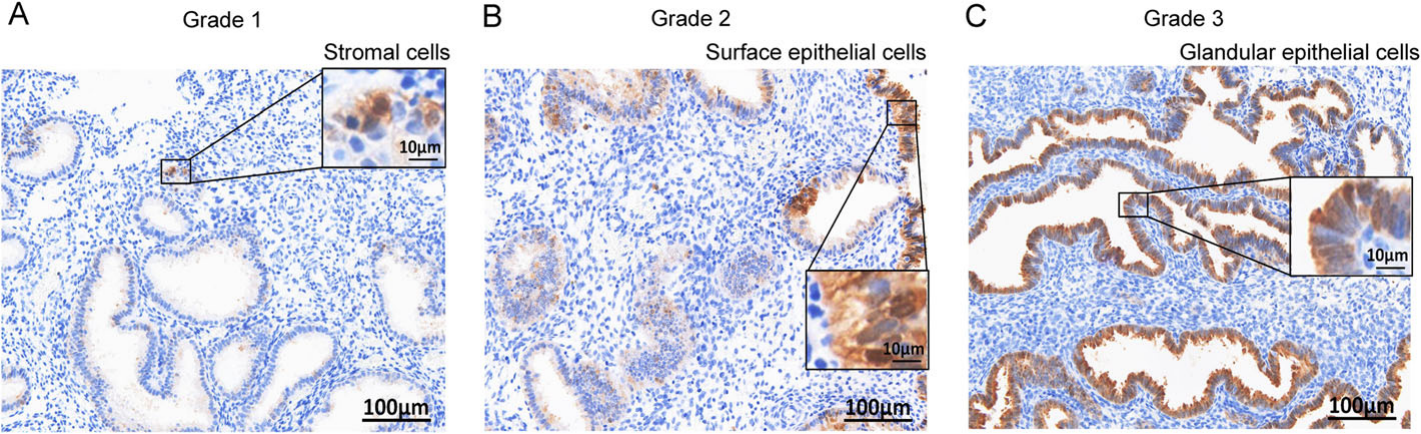
Immunohistochemical grading of the expression of IDO in endometrium. The grading was performed based on staining intensity, as described in the materials and methods, grade 1: no or weak staining and scarcely positive, IDO was localized in the stromal compartment (**a**); grade 2: moderately stained and focally positive, IDO was localized in surface epithelial cells (**b**); grade 3: strongly stained and diffusely positive, IDO was localized in glandular epithelial cells (**c**); scale bars = 100 µm, with insets = 10 µm

Based on immunohistochemical staining, IDO was mainly localized in surface epithelial cells, glandular epithelial cells and a small number of cells within the stromal compartment (including stromal cells and leukocytes) in endometrium (Fig. 2). According to the previous studies, immunostaining grading scale was used to evaluate the expression levels of IDO in all the patients included.

### Population characteristics

During the study period, 140 couples receiving their first IVF-ET treatments formed the present study population, including 36 women with grade 1 endometrial IDO immunohistochemical staining, 46 women with grade 2 endometrial IDO immunohistochemical staining, and 58 women with grade 3 endometrial IDO immunohistochemical staining.

Table 1 depicts patient and IVF cycle characteristics by pregnancy outcome. Clinical pregnancy rate was 64.3% and live birth rate was 57.1% in our study population. Baseline patient characteristics including age, BMI, baseline serum FSH level, infertility duration and diagnosis were compared among the different groups. No significant differences were noted. Clinical pregnancy was associated with lower dose of gonadotropin used (*P* = 0.046), whereas live birth was associated with shorter duration of gonadotropin stimulation (*P* = 0.039).

**Table 1:** Clinical characteristics of infertile women during their first IVF treatment cycle by pregnancy outcome

Maternal characteristics	Pregnant (***n***=90)	Not pregnant (***n***=50)	***P***-value	Live birth (***n***=80)	No live birth (***n***=60)	***P***-value
**Maternal age (y)**	31.7 ± 3.9	32.3 ± 4.6	0.417	31.9 ± 3.9	32.0 ± 4.6	0.866
**Maternal BMI (kg/m**^**2**^**)**	21.5 (19.9-23.4)	20.6 (19.2-23.4)	0.220	21.5 (19.9-23.3)	20.7 (19.3-23.4)	0.366
**Type of infertility**			0.207			0.261
Primary	46 (51.1%)	20 (40.0%)		41 (51.3%)	25 (41.7%)	
Secondary	44 (48.9%)	30 (60.0%)		39 (48.7%)	35 (58.3%)	
**Infertility duration (y)**	3.0 (1.0-4.0)	2.0 (1.0-3.0)	0.265	3.0 (1.0-4.0)	2.0 (1.0-3.0)	0.226
**Baseline FSH level (IU/L)**	5.5 (4.2-6.8)	6.3 (4.6-7.0)	0.169	5.5 (4.2-6.7)	6.3 (4.8-7.1)	0.058
**Duration of gonadotropin stimulation (d)**	9.0 (8.0-10.0)	10.0 (9.0-10.3)	0.083	9.0 (8.0-10.0)	10.0 (9.0-10.0)	0.039
**Total dose of gonadotropin (IU)**	2263.3 ± 694.7	2504.3 ± 650.0	0.046	2292.7 ± 692.8	2425.0 ± 676.3	0.260
**Serum E**_**2**_**level (pg/mL) on hCG day**	2461.5 (1766.5-3785.0)	1998.0 (1528.3-2834.5)	0.116	2510.0 (1793.0-3902.5)	1998.0 (1566.8-2806.5)	0.063
**EMT (mm) on hCG day**	11.0 (10.0-13.0)	11.0 (10.0-13.0)	0.406	11.0 (10.0-13.0)	11.0 (10.0-12.8)	0.711
**No. of oocyte retrieved**	13.0 (10.0-15.0)	11.5 (8.8-15.3)	0.248	13.0 (10.3-15.8)	12.5 (9.0-15.0)	0.177
**No. of fertilized occytes**	10.0 (7.8-13.0)	9.0 (7.0-12.3)	0.419	10.0 (8.0-13.0)	9.0 (7.0-12.0)	0.255
**No. of embryos transferred**	2 (1-2)	2 (1-2)	0.836	2 (1-2)	1 (1-2)	0.160
**Cycles with different technologies**			0.932			0.956
IVF	66 (73.3%)	37 (74.0%)		59 (73.8%)	44 (73.3%)	
ICSI	24 (26.7%)	13 (26.0%)		21 (26.2%)	16 (26.7%)	
**Embryo type**			0.180			0.640
Cleavage embryo	26 (28.9%)	20 (40.0%)		25 (31.3%)	21 (35.0%)	
Blastocyst	64 (71.1%)	30 (60.0%)		55 (68.7%)	39 (65.0%)	
**Embryo quality**			0.134			0.074
Cycle with high-quality embryos	87 (96.7%)	45 (90.0%)		78 (97.5%)	54 (90.0%)	
Cycles without high-quality embryos	3 (3.3%)	5 (10.0%)		2 (2.5%)	6 (10.0%)	

Note: *BMI* body mass index, *E*_*2*_ estradiol, *EMT* endometrial thickness, *IVF* in vitro fertilization, *ICSI* intracytoplasmic sperm injection, *hCG* human chorionic gonadotropin

Values are numbers (percentages) of cases, mean ± standard deviation or median (interquartile range)

Continuous variables: Mann-Whitney *U*-test or independent *t*-test; categorical variables: Chi-square test or Fisher’s exact test

### Association of IDO expression and pregnancy outcomes

**Fig. 3:**
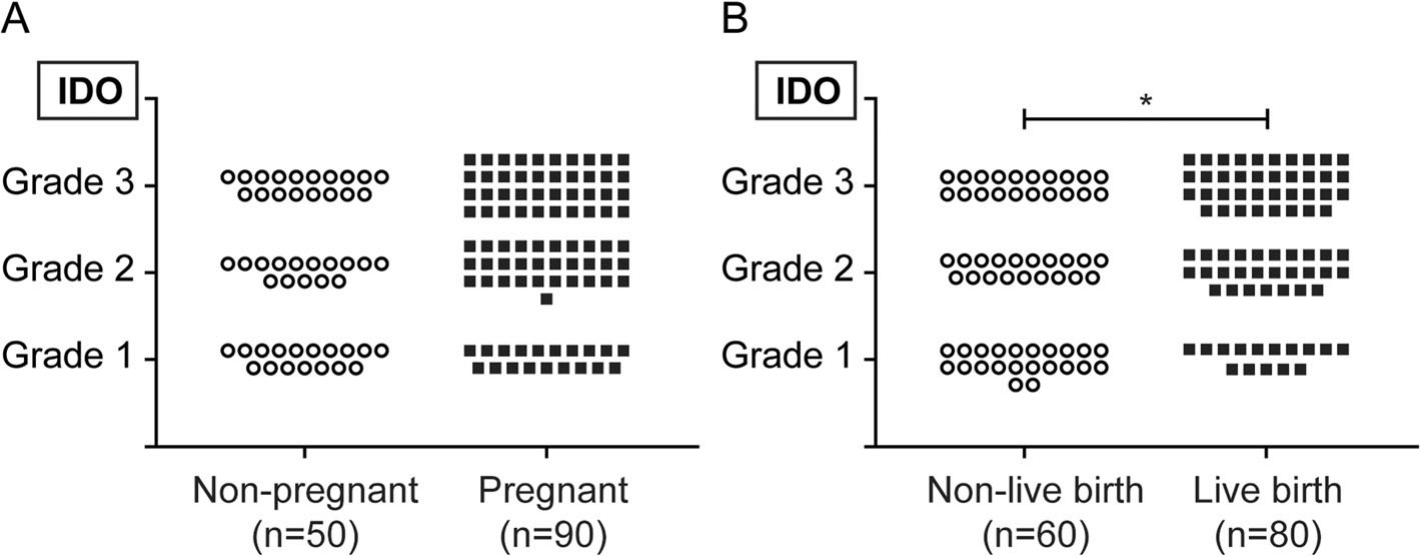
Comparison of endometrial IDO expression between different groups. **a** The statistical chart showing the expression level of IDO in endometrium between the non-pregnant group (*n* = 50) and pregnant group (*n* = 90). **b** The statistical chart showing the expression level of IDO in endometrium between the non-live birth group (*n* = 60) and live birth group (*n* = 80). *P < 0.05, Mann-Whitney *U* test

There was no significant difference on the endometrial IDO expression level between women who had clinical pregnancy and those who failed (*P* > 0.05). However, the endometrial IDO expression level was significantly higher among women who had live birth compared with those who had no live birth (*P* = 0.031) (Fig. 3).

A binary logistic regression analysis was performed to analyze the association between endometrial IDO expression and live birth (Table 2). As indicated in Table 2, more live births were positively correlated with higher endometrial IDO expression. After adjusted for maternal age and BMI, women with grade 3 IDO expression still had a higher live birth rate (aOR 2.857, 95% CI 1.184–6.891, *P* = 0.019). Moreover, a secondary analysis was performed including age, BMI and duration of gonadotropin stimulation in the model, which did not substantially alter the estimates, live birth remained positively associated with an increased endometrial IDO expression (aOR 2.863, 95% CI 1.180–6.947, *P* = 0.020).

**Table 2:** Logistic regression for the association between endometrial IDO expression level and live birth rate of patients undergoing first IVF treatment

Variables	OR (95% CI)	***P***-value	Adjusted OR (95% CI)^**a**^	***P***-value^**a**^	Adjusted OR (95% CI)^**b**^	***P***-value^**b**^
**IDO expression level**						
Grade 1	Ref	Ref	Ref	Ref	Ref	Ref
Grade 2	1.989 (0.821-4.821)	0.128	2.090 (0.846-5.165)	0.110	2.199 (0.880-5.491)	0.092
Grade 3	2.660 (1.130-6.259)	0.025	2.857 (1.184-6.891)	0.019	2.863 (1.180-6.947)	0.020

^a^Adjusted for maternal age and BMI

^b^Adjusted for maternal age, BMI and duration of gonadotropin stimulation

## Discussion

In the present study, we found that endometrial IDO expression was positively associated with more live birth among women during their first IVF treatment, after adjusting for important confounders. Our results suggest that IDO might play a functional role in predicting live birth among women in their first IVF treatment.

A successful pregnancy depends on the regulation of maternal immune system at the maternal-fetal interface to enable a functional placenta to develop. Thus, the semiallogenic fetus is tolerated by the mother and protected by a very efficient process of immune tolerance during the entire process. Since Munn et al. [10] formulated the hypothesis that the expression of IDO at the maternal-fetal interface could prevent immunological rejection of the fetal allograft, there have been many studies on the physiological significance of IDO in human pregnancy. The role of IDO in regulating maternal-fetal tolerance is attributed to its immunosuppressive properties, which are based on inhibition of T cell proliferation through deletion of the essential amino acid tryptophan since T cells exhibit a tryptophan-sensitive G1 cell cycle arrest [20]. Our previous study showed that the expression level of IDO was downregulated in RM patients compared with controls, suggesting an important role of IDO in the maintenance of normal pregnancy [18]. Furthermore, the production of IFN-γ, which is an important and potent stimulator of IDO expression, is found to be significantly decreased in RM patients, which may lead to the reduction in IDO and its activity [21]. Previous studies have demonstrated a link between IDO and preeclampsia [19]. IDO expression was significantly downregulated in preeclampsia placenta, and the reduction in IDO expression was associated with the severity of maternal hypertension and proteinuria. Thus, IDO may play an important role in maintaining a healthy pregnancy.

In our study, endometrial IDO expression starts in the mid-luteal phase around the time when embryo implantation is expected. Thus, this early IDO expression at maternal-fetal interface may be considered as preparation of the endometrium for maternal establishment for fetal tolerance and the regulation of trophoblast invasion.

It is unclear why we failed to see any relationship between IDO expression levels and clinical pregnancy and miscarriage rates. This result can be partially explained by the recent postulation that the first trimester of pregnancy is associated with inflammation, which is required for blastocyst implantation [22]. Pregnancy can be divided into three distinct immunological processes characterized by different biological processes. During the first stage, the blastocyst has to break through the epithelial lining of the uterus and damage the endometrial tissue in order to implant; then the trophoblast must replace the endothelium and vascular smooth muscle of the maternal blood vessels to secure an adequate placental-fetal blood supply. Thus, the first trimester of pregnancy can be viewed as a pro-inflammatory phase. Consistently, higher IDO expression was not associated with higher clinical pregnancy rate and lower miscarriage rate in our study.

We are the first study demonstrating the relationship between endometrial IDO expression and pregnancy outcomes in infertile patients seeking IVF-ET treatment. Although we have concluded that endometrial IDO expression was positively correlated with live birth, several limitations should be further considered. First, this is a retrospective study, so a well-designed prospective study should be conducted to assess the prognostic role of IDO. Second, we cannot exclude the interference of progesterone to the relationship between IDO and pregnancy outcome, as progesterone was used as a routine method to provide luteal phase support in our center. Third, our study did not include frozen-thawed ET cycles, which may induce some unknown bias. Last, well-designed clinical studies are warranted to elucidate the pathogenesis of IDO expression and its subsequent effects on IVF success.

## Conclusions

In conclusion, our data suggest that an elevated level of endometrial IDO expression is associated with an increased rate of live birth in IVF treatment. The endometrial IDO expression may also be a new therapeutic target for better IVF treatment, however, further large prospective study are required to verify this hypothesis.

## Data Availability

The datasets used and/or analyzed during the current study are available from the corresponding author on reasonable request.

## Abbreviations

IDO: Indoleamine 2,3-dioxygenase
IVF-ET: In vitro fertilization and embryo transfer
RM: Recurrent miscarriage
COH: Controlled ovarian hyperstimulation
PCOS: Polycystic ovary syndrome
PBS: Phosphatebuffered saline
hCG: Human chorionic gonadotropin
ICSI: Intracytoplasmic sperm injection
BMI: Body mass index
E_2_: Estradiol
EMT: Endometrial thickness
